# Microfluidic-Derived Docosahexaenoic Acid Liposomes for Targeting Glioblastoma and Its Inflammatory Microenvironment

**DOI:** 10.1021/acsami.4c01368

**Published:** 2024-07-23

**Authors:** Daniel Mendanha, Marta R. Casanova, Sara Gimondi, Helena Ferreira, Nuno M. Neves

**Affiliations:** † 3B’s Research Group, I3Bs—Research Institute on Biomaterials, Biodegradables and Biomimetics, University of Minho, Headquarters of the European Institute of Excellence on Tissue Engineering and Regenerative Medicine, AvePark, Parque de Ciência e Tecnologia, Zona Industrial da Gandra, 4805-017 Barco, Guimarães, Portugal; ‡ ICVS/3B’s-PT Government Associate Laboratory, 4805-017 Barco, Braga/Guimarães, Portugal

**Keywords:** docosahexaenoic acid, glioblastoma, inflammation, liposome, microfluidic, pro-inflammatory mediators

## Abstract

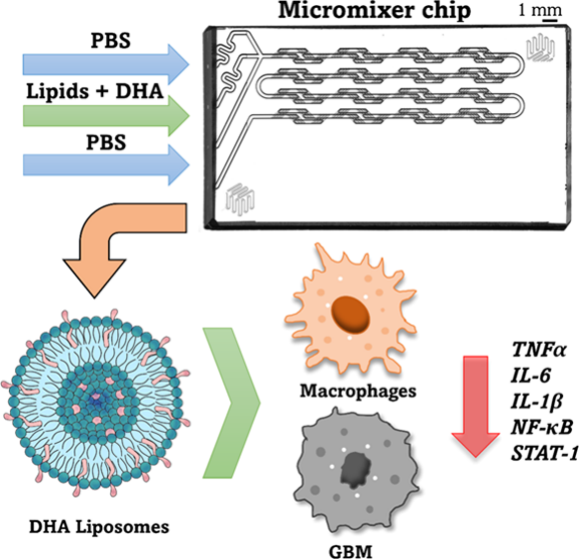

Glioblastoma (GBM) is the most common malignant primary brain tumor, characterized by limited treatment options and a poor prognosis. Its aggressiveness is attributed not only to the uncontrolled proliferation and invasion of tumor cells but also to the complex interplay between these cells and the surrounding microenvironment. Within the tumor microenvironment, an intricate network of immune cells, stromal cells, and various signaling molecules creates a pro-inflammatory milieu that supports tumor growth and progression. Docosahexaenoic acid (DHA), an essential ω3 polyunsaturated fatty acid for brain function, is associated with anti-inflammatory and anticarcinogenic properties. Therefore, in this work, DHA liposomes were synthesized using a microfluidic platform to target and reduce the inflammatory environment of GBM. The liposomes were rapidly taken up by macrophages in a time-dependent manner without causing cytotoxicity. Moreover, DHA liposomes successfully downregulated the expression of inflammatory-associated genes (*IL-6*; *IL-1β*; *TNFα*; *NF-κB*, and *STAT-1*) and the secretion of key cytokines (IL-6 and TNFα) in stimulated macrophages and GBM cells. Conversely, no significant differences were observed in the expression of *IL-10*, an anti-inflammatory gene expressed in alternatively activated macrophages. Additionally, DHA liposomes were found to be more efficient in regulating the inflammatory profile of these cells compared with a free formulation of DHA. The nanomedicine platform established in this work opens new opportunities for developing liposomes incorporating DHA to target GBM and its inflammatory milieu.

## Introduction

Glioblastoma (GBM) is the most aggressive and common malignant form of brain cancer globally, affecting approximately 3 individuals per 100,000 annually.^1,2^ It is characterized by rapid tumor growth, high invasiveness into surrounding brain tissue, intra- and intertumoral heterogeneity, and abnormal inflammation.^3−5^ Moreover, the presence of biological barriers, namely, the blood–brain barrier (BBB)^6^ and blood–tumor barrier (BTB),^7^ creates a unique tumor microenvironment (TME) in the cancer field, explaining the low efficiency of therapies for this cancer type. Indeed, the uniqueness and localization of these tumors render the development of effective treatments challenging, being the standard therapeutic approach virtually unchanged since 2005.^8^ This treatment, consisting of the GBM maximal surgical resection followed by a chemotherapy and radiotherapy dose schedule, only improves the patient’s overall survival by a few months.^8,9^

The highly immunosuppressive and inflammatory microenvironment of GBM are key hallmarks of these tumors, largely contributing to its aggressiveness and resistance to therapies.^10−12^ Relatively to other tumor types, GBM presents a large number of myeloid cells (*e.g.*, macrophages and neutrophils) but a reduced percentage of tumor-infiltrating lymphocytes and other immune effector cells.^10,13^ The abnormal balance between the two immunomodulatory cell types in conjugation with the secreted immunosuppressive molecules by cancer cells, explains the current challenges in immunotherapy strategies for GBM.^13^ Moreover, the panoply of inflammatory mediators present in the TME (*e.g.*, cytokines, chemokines, and growth factors) generates an inflammatory network at the tumor site, promoting tumor progression, growth, and invasion.^14−16^ As previously referred, macrophages are an abundant cell type in the TME that presents a high plasticity, changing their phenotype and role in response to different stimuli.^17,18^ The macrophage’s migration to the tumor site occurs in response to chemoattractants released by cancer cells, such as monocyte chemoattractant proteins-1 (MCP-1) and -3 (MCP-3), and colony-stimulating factors-1 (CSF-1) and -2 (CSF-2). However, upon arrival at the tumor site, they change their phenotype to tumor-associated macrophages (TAMs).^18,19^ The constant cross-talk between TAMs and GBM cells leads to the release of several interleukins (IL; *e.g.*, IL-6 and IL-1β) and growth factors (*e.g.*, epidermal growth factor—EGF and transforming growth factor β—TGF-β) by these myeloid cells, providing a favorable environment for the tumor progression.^18^ Moreover, cancer cells secrete several inflammatory mediators, including IL-1β, IL-6, and tumor necrosis factor-α (TNFα) to the TME that also promote carcinogenesis.^20^ Considering these features, some strategies have been developed to target TAMs and the inflammatory environment of GBM. These strategies can in a simplistic way be split into three categories: the inhibition of TAM recruitment, the killing of TAMs, or the re-education and change in the phenotype of these immune cells.^20,21^

Docosahexaenoic acid (DHA) is an omega-3 (ω3) polyunsaturated fatty acid that has been investigated as a potential therapeutic agent for GBM.^22^ Indeed, this essential nutrient with a fundamental role in brain development and function^23^ can induce GBM cell death through apoptosis and autophagy.^24^ DHA can also inhibit their proliferation and migration of cancer cells by the activation of protein kinase C pathways in a fatty acid-binding protein (B-FABP)-dependent manner.^25^ In addition to its effects on GBM cells, DHA has been shown to effectively reduce the inflammatory scenario in a variety of diseases, such as rheumatoid arthritis, inflammatory bowel diseases, asthma, and cancer.^26^ Indeed, the bioactive metabolic derivatives of DHA, including resolvins, protectins, and oxylipins, exert strong anti-inflammatory properties.^27^ Additionally, enriched diets in ω3 fatty acids, like DHA, were able to reduce tumor-associated inflammatory cytokines, like IL-6, IL-10, and TNFα in prostate cancer.^28^ Moreover, in this cancer type, DHA treatment showed the capacity to induce a local anti-inflammatory response.^29^ Another key aspect of DHA is its ability to cross the BBB and mediate the targeted delivery of bioactive agents into the brain.^30^ This unique characteristic opens the possibility of using this fatty acid for the targeting of brain diseases or as a coadjuvant therapy agent.

Considering these findings, in this work, a new therapeutic formulation for GBM based on DHA was developed to target not only the cancer cells but also the surrounding inflammatory microenvironment. To overcome DHA’s poor hydrosolubility and susceptibility to degradation, it was included in liposomes (DHA liposomes) produced through a microfluidic system. Microfluidics for liposome production offers several advantages, such as precise control over experimental parameters, high reproducibility and yield, as well as reduced synthesis time.^31^ Indeed, it can be used to produce liposomes of clinically relevant standards.^31,32^ After DHA liposomes characterization, the *in vitro* potential of the developed liposomes in the regulation of macrophages and the GBM inflammatory profile was assessed. For that, the targeting and internalization of cytocompatible concentrations of liposomes by macrophages over time was assessed. Indeed, this study focused on modulating the inflammatory behavior of stimulated macrophages, instead of eliminating them. To reveal the power of the developed formulation, the expression of key genes and cytokines associated with an inflammatory scenario in stimulated macrophages and GBM cells was performed. Thus, this strategy aims to overcome the drawbacks of administering free ω3 fatty acids by increasing the bioavailability and concentration of these active molecules in the inflammatory TME of GBM.

## Materials and Methods

### Reagents

Dulbecco’s modified Eagle’s medium (DMEM; Sigma, D5523), fetal bovine serum (FBS; Gibco, A31608), and trypLE Express (Gibco, 12605) were purchased from Life Technologies (Carlsbad, CA). Roswell Park Memorial Institute 1640 (RPMI 1640) medium (Gibco, 22400), l-α-phosphatidylcholine (PC; Sigma, P3556), cis-4,7,10,13,16,19-docosahexaenoic acid (DHA; Sigma, D2534), cholesterol (Sigma, C8667), lipopolysaccharide (LPS; Sigma, L8274), phorbol 12-myristate 13-acetate (PMA; Sigma, P8139), phalloidin–tetramethyl rhodamine B isothiocyanate (phalloidin-TRITC; Sigma, P1951), and phosphate buffer solution (PBS; Sigma, P4417) were purchased from Sigma-Aldrich (St. Louis, MI). Quant-iT PicoGreen dsDNA Assay Kit (Thermo Fisher Scientific, P7589), ethanol (99.8%; Thermo Fisher Scientific, E/0650DF/C17), and NBD cholesterol (22-(*N*-(7-nitrobenz-2-oxa-1,3-diazol-4-yl)amino)-23,24-bisnor-5-cholen-3β-Ol; Invitrogen, N1148) were bought from Thermo Fisher (Waltham, MA). Recombinant human interferon-γ (IFN-γ; Abcam, ab9659) was acquired from Abcam (Cambridge, U.K.). The Deep Blue Cell Viability Kit (Biolegend, 424702) was obtained from BioLegend (San Diego, CA). LabAssay Phospholipid (FUJIFILM Wako, LABPLIP-M1) was purchased from FUJIFILM Wako (Osaka, Japan), and Mixer Chip Part #3200401 was purchased from Dolomite (Royston, U.K.). 4′,6′-Diamino-2-Fenil-indol (DAPI; Biotiumn, 40009) was purchased from Biotiumn (California).

### Cell Lines and Culture Conditions

The human GBM cell line U87 (ATCC HTB-14) and the human leukemia monocytic cell line (THP-1; ATCC TIB-202) were cultured in DMEM and RPMI 1640 media, respectively, supplemented with 10% FBS and 1% penicillin-streptomycin at 37 °C in a humidified 5% (v/v) CO_2_ atmosphere.

Monocytes were differentiated into macrophages (Mφ) with 100 nM PMA for 24 h, followed by a 48 h rest period under the described culture conditions. To promote the release of inflammatory mediators, macrophages were stimulated with LPS (100 ng/mL) and IFN-γ (20 ng/mL), as previously reported,^33,34^ and designated as stimulated macrophages (stimulated Mφ).

### Liposomes Synthesis

In this study, liposomes were synthesized by using a microfluidic system. Particularly, a micromixer chip designed for the millisecond mixing of three fluid streams through 12 mixing stages of microsized channels (internal cylindric channel cross section of 125 μm × 350 μm and 50 μm depth × 125 μm width) was used as previously reported.^35^ Briefly, the experimental setup consisted of three syringes controlled by a single syringe pump (New Era Pump Systems; N300; NY) and a double-syringe pump (Kranalytical; FUSION 200; U.K.) connected to the chip through the H interface and the linear connector 4-way. The ethanolic solution (15 mM) of PC, cholesterol, and DHA at a 1:0.5:0.5 molar ratio was loaded in one syringe and pumped through the middle channel to synthesize DHA liposomes. For control (CTR) liposomes, an ethanolic solution of 15 mM without DHA was used at a molar ratio of 1.5:0.5 of PC and cholesterol, respectively. The other two syringes in the double-syringe pump were loaded with PBS to enter the chip via the side channels. The flow rates of 125 and 250 μL/min for the lipid and PBS solutions, respectively, were kept constant during liposome synthesis.

After liposome synthesis, the suspensions were placed in a rotatory evaporator for 10 min at 50 mbar to evaporate the organic solvent. Regarding liposome uptake assays, NBD cholesterol was added at 1% of the total amount of cholesterol in the lipid formulation before liposome synthesis. The removal of free DHA from liposome suspensions was obtained by size exclusion chromatography using PD-10 desalting columns (GE Healthcare), according to the manufacturer’s instructions.

### Liposomes Characterization

The hydrodynamic size and polydispersity index (PDI) of the synthesized liposomes were evaluated by dynamic light scattering (DLS) using disposable cuvettes, at 25 ± 0.1 °C, in Malvern Zetasizer NS (Malvern Instruments, U.K.) equipment. The surface potential (ζ-potential) of the liposomes was analyzed by laser Doppler microelectrophoresis with a dip cell, in the same equipment. The samples were diluted in PBS to obtain a final concentration of 750 μM, and the measurements were performed at 25 °C at a refractive index of 1.330, a dielectric constant of 79.0, and a viscosity of 0.8882 cP.

The DHA concentration within the liposomes was determined through high-performance liquid chromatography (HPLC; Alliance 2695), as previously reported.^36^ Briefly, the stationary phase employed was an sb-c18 column (Zorbax), while the gradient mobile phase was composed of a mixture of acetonitrile (86:100%) and 0.5% phosphoric acid (0–14%). Standards and samples were prepared in 0.2% acetic acid; a volume of 10 μL was injected, and the flow rate was 1 mL/min for a run time of 30 min. The column temperature was maintained at 4 °C; the detection was monitored at a wavelength of 205 nm, and the DHA concentration in the samples was inferred from the standard curve obtained.

The morphology of the liposomes was evaluated by atomic force microscopy (AFM). The samples were diluted with HEPES buffer at a final concentration of 150 μM and a drop of 10 μL was placed on top of a glass slide and left to air-dry. The AFM images were acquired with a JPK NanoWizard 3 (Bruker) in AC mode in air with AFM cantilevers (ACTA, AppNano) made of silicon. A spring constant between 13 and 77 N/m and a frequency between 200 and 400 kHz were used during image capture.

### Cell Metabolic Activity and DNA Concentration

To address the toxicity of CTR liposomes and DHA liposomes toward macrophages, the metabolic activity after treatment was determined using the Alamar blue assay, following the manufacturer’s instructions. Briefly, 2.5 × 10^6^ macrophages were seeded and incubated with several concentrations of DHA liposomes (0, 25, 50, 100, and 150 μM) or lipid concentrations (0, 125, 250, 500, and 1000 μM) for 1, 2, or 3 days. At different time points, the samples were incubated for 4 h with a medium containing 10% Alamar blue. The fluorescence was measured in a microplate reader (Synergy HT, BioTek), using an excitation wavelength of 530 nm and an emission wavelength of 590 nm.

The DNA concentration of macrophages after DHA liposomes incubation for 3 days was analyzed using a dsDNA quantification kit (Quant-IT PicoGreen), according to the manufacturer’s instructions. Briefly, the fluorescence of the samples was measured in a microplate reader (Synergy HT, BioTek), using an excitation wavelength of 485 nm and an emission wavelength of 530 nm. DNA concentration of the samples was inferred from the standard curve obtained.

After subtracting the blank fluorescence from the sample fluorescence values, the metabolic activity and DNA concentration were normalized toward the values of the control (cells without treatment) and expressed in percentage.

### Liposomes Cellular Uptake

To assess the uptake of liposomes containing or not DHA by macrophages and stimulated macrophages, confocal microscopy, and flow cytometry analyses were conducted.^37^ For confocal microscopy, cells were seeded into μ-slide well chambers (Ibidi, Germany) at a concentration of 4 × 10^4^ cells, after which they were treated with 500 μM of the respective fluorescently labeled liposomes (CTR and DHA liposomes) for 4 h. The cells were then fixed with 4% paraformaldehyde (PFA) for 30 min, washed with PBS, and subsequently incubated with phalloidin (1:200; #P1951) and DAPI (1:1000, #40009) for 30 and 5 min, respectively, before being washed with PBS. Confocal microscopy analyses were carried out in a confocal laser scanning microscope (TCS SP8, Leica).

The percentage of positive cells after liposome treatment was determined by flow cytometry. Briefly, after 24 h of cell seeding, 500 μM liposomes were added to the cells and incubated for 1, 4, and 24 h. The cells were then washed with PBS, collected with PBS-EDTA (10 mM), fixed, and analyzed. A total of 20,000 events were acquired per condition by a BD FACSCalibur flow cytometer (Biosciences, NJ), and the results were analyzed using the FlowJo 10 software.

### RNA Isolation and Real-Time Quantitative Polymerase Chain Reaction

Macrophages and U87 cells were incubated for 1, 2, or 3 days with either free DHA or DHA liposomes at a concentration of 50 μM. The corresponding lipid concentration of empty liposomes was used as a control (CTR liposomes). Cells cultured only in the presence of a medium (without the addition of liposomes with or without DHA) were also used as controls. The expression of several genes related to inflammatory mediators was analyzed through Real-Time Quantitative Polymerase Chain Reaction (RT-qPCR).^33^ Following the incubation period, macrophages and GBM cells were washed with PBS and stored at −80 °C until further use. The RNA extraction was performed using Tri reagent (Life Science) according to the manufacturer’s instructions. The concentration and purity of RNA were determined by NanoDrop ND-100 Spectrophotometer (NanoDrop Technologies Inc.) analyses (Table S1). The complementary DNA (cDNA) was synthesized from 100 ng of total RNA through reverse transcription using a qScript DNA synthesis kit (Quanta Biosciences, VWR), following the manufacturer’s instructions.

The amplification and quantification of inflammation mediator genes (Table 1) were carried out by the PerfeCtaTM SYBR Green system (Quanta Biosciences, VWR, Netherlands), and the qPCR reactions were carried out in a Mastercycler ep Gradient S realplex thermocycler (Eppendorf, Germany). The expression of the target genes was normalized by using glyceraldehyde-3-phosphate dehydrogenase (*GAPDH*) as the reference gene. The Livak method (2^–ΔΔCT^ method) was used to analyze the gene expression and quantification, with the expression obtained in the control conditions (macrophages and GBM cells only in culture medium) serving as the calibrators.

**Table 1:** Primer Sequences, Primary NCBI References, Cycle Number, Annealing Temperature, and Product Size Used for the RT-qPCR Procedures^a^

gene	forward (5′-3′)	reverse (5′-3′)	NCBI refs	cycle number	annealing temperature (°C)	product size (bp)
*GAPDH*	CAACTCCCTCA	GGCATGGACT	gene ID: 2597	35	56.3	118
	AGATTGTCAGCAA	GTGGTCATGA				
*TNFα*	ATGTTGTAGCAA	TGATGGCAGAG	gene ID: 7124	35	59	249
	ACCCTCAAGC	AGGAGGTTG				
*IL-6*	AGGAGACTTG	GCATTTGTGG	gene ID: 3569	35	59	196
	CCTGGTGAAA	TTGGGTCAG				
*IL-1β*	TGAGCTCGCC	AGGAGCACTTC	gene ID: 3553	35	59	92
	AGTGAAATGA	ATCTGTTTAGGG				
*NF-Kβ*	GGGTAACTCTG	GCTATTGCTATC	gene ID: 4790	35	60	147
	TTTTGCACCTA	ATGGCTAGA				
*STAT-1*	GATCTCCAAC	GCACATGGTG	gene ID: 6772	35	60	108
	GTCAGCCAGC	GAGTCAGGAA				
*IL-10*	AAGACCCAG	AATCGATGACA	gene ID: 3586	35	60	85
	ACATCAAGGCG	GCGCCGTAG				

a GAPDH = glyceraldehyde-3-phosphate dehydrogenase; TNFα = tumor necrosis factor-α; IL-6 = interleukin 6; IL-1β = interleukin 1 β; NF-κB = nuclear factor kappa-light-chain-enhancer of activated B cells; STAT-1 = signal transducer and activator of transcription 1; IL-10 = interleukin 10.

### Quantification of Secreted Cytokines

To assess the effects of different liposome formulations on the secretion of inflammatory cytokines, macrophages and GBM cells were treated with CTR liposomes, free DHA, and DHA liposomes for 3 days. Cells without the addition of liposomes were used as basal conditions (controls). For macrophages, both stimulated and nonstimulated macrophages cultured in the absence of any formulation to test served as controls. The culture supernatants were collected and stored at −80 °C until use. The concentrations of IL-6 and TNFα in the supernatants were quantified by commercially available ELISA kits (R&D Systems, Minneapolis, MN), following the recommendations of the manufacturer. Briefly, a 96-well plate was coated with the respective capture antibody overnight at room temperature. After blocking with 1% bovine serum albumin in PBS for 1 h, 100 μL of culture supernatants or standards were added to each well and incubated for 2 h at room temperature. The plate was then washed, and the respective detection antibody was added, being this mixture incubated for 2 h, at room temperature. After washing, the plate was incubated with streptavidin conjugated to horseradish peroxidase for 20 min, and then with substrate solution for 20 min, at room temperature. The reaction was stopped with the addition of the stop solution, and the absorbance was measured at 450 nm using a microplate reader (Synergy HT, BioTek). The concentration of cytokines in each sample was determined by interpolation using a standard curve of absorbance versus concentration.

### Statistical Analyses

Statistical analyses were performed using GraphPad Prism 8 software (GraphPad Software, Inc., San Diego). To compare two or more groups at different time points and conditions, a two-way ANOVA was employed followed by Tukey’s or Dunnett’s multiple comparison tests. The results are expressed as mean ± standard deviation (SD) or mean ± standard error of mean (SEM), of three independent experiments, and statistical significance was set as *p* < 0.05 for a 95% confidence interval.

## Results

**Figure 1:**
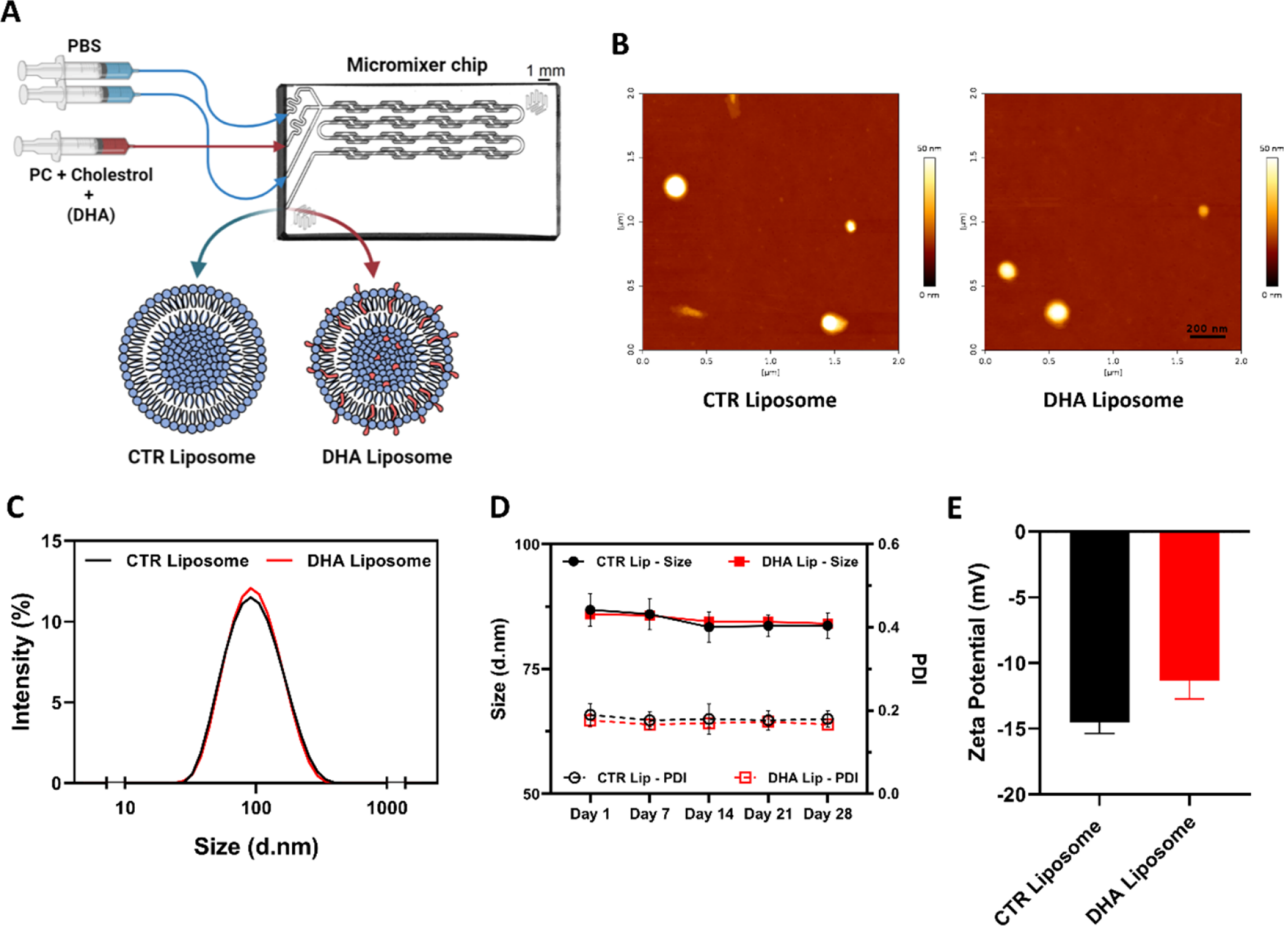
Schematic illustration of liposome synthesis and their physicochemical characterization. Synthesis representation of liposomes without (control; CTR liposome) and with docosahexaenoic acid (DHA; DHA liposome) through a micromixer chip device (PC, phosphatidylcholine; PBS, phosphate-buffered saline) (A). Atomic force microscopy (AFM) images of CTR and DHA liposomes (B). Intensity size distribution graph of CTR and DHA liposomes (C) and stability assessment throughout size and polydispersity index (PDI) measurements for 28 days of (D). Surface charge of liposome nanosuspensions (E).

The liposome synthesis was achieved through a micromixer device that allowed for stable and uniform mixing of the organic stream with the two aqueous streams at a constant flow rate (Figure 1A). We assessed if the inclusion of DHA in the liposome composition led to a change in their physicochemical characteristics. As can be observed by AFM images (Figure 1B), the presence of DHA did not change the spherical-like morphology of the developed nanostructures. Moreover, CTR and DHA liposomes presented identical hydrodynamic sizes (86.87 ± 3.25 and 86.01 ± 0.07 nm, respectively; Figure 1C). The developed homogeneous suspensions (PDI < 0.2) of liposomes were also stable for at least 28 days (size variations below 4 nm; Figure 1D). The surface electrical charge of the liposome membranes was negative independently of the presence of DHA in the liposome’s composition, with ζ-potentials of −14.53 ± 0.84 and −11.35 ± 1.39 mV, for CTR and DHA liposomes, respectively (Figure 1E).

**Figure 2:**
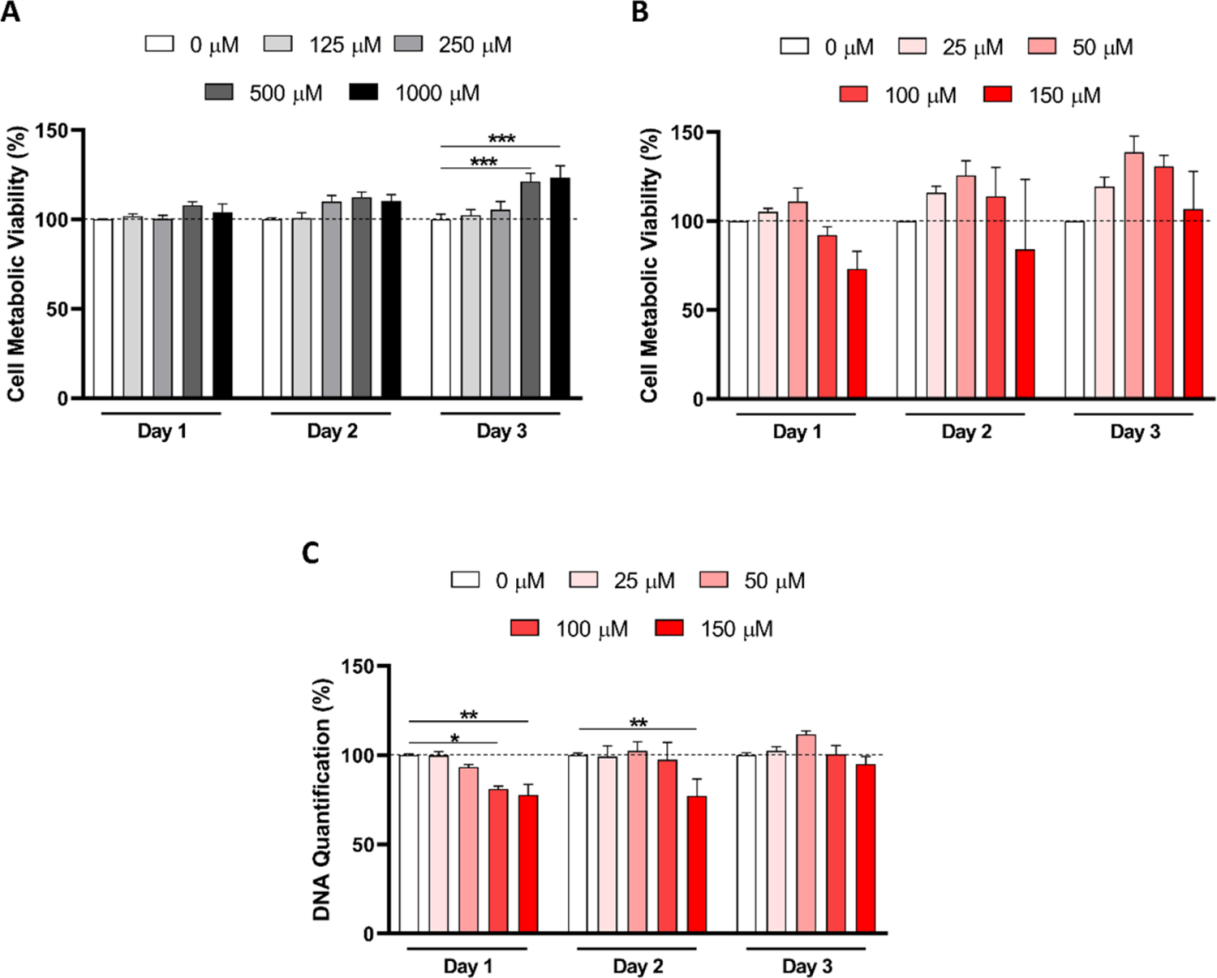
Cytotoxicity of control (CTR) and docosahexaenoic acid (DHA) liposomes. Metabolic activity of stimulated macrophages (A) assessed by Alamar blue assay after 1, 2, and 3 days of exposure to CTR liposomes (0, 125, 250, 500, and 1000 μM). Metabolic activity (B) and DNA quantification (C) of stimulated macrophages incubated with DHA liposomes (0, 25, 50, 100, and 150 μM DHA) expressed relative to the control condition (0 μM liposomes). Data was analyzed by 2-way ANOVA, and *(*p* < 0.05), **(*p* < 0.01), and ***(*p* < 0.001) were used to denote significant differences between groups.

After the characterization, the cytocompatibility of the developed formulations was evaluated. First, the metabolic activity of stimulated macrophages was assessed in the presence of several concentrations of CTR liposomes (from 125 to 1000 μM). As can be observed in Figure 2A, no significant differences were found between cells cultured or not in the presence of liposomes on day 1 and day 2. After 3 days of incubation, the conditions with the highest tested concentrations of liposomes led to an increase in the metabolic activity of stimulated macrophages (21.18 and 23.47% for 500 and 1000 μM, *p* < 0.001, respectively; Figure 2A). To assess the effect of DHA in stimulated macrophages, the cells were incubated for 3 days with liposomes containing concentrations of this ω3 fatty acid ranging from 25 to 150 μM (Figure 2B). No significant differences were observed between the control condition and the conditions incubated with DHA liposomes. However, with regard to the DNA quantification of the cells exposed to DHA liposomes, a significant decrease in DNA content was observed at day 1 for the 100 μM (*p* = 0.017) and 150 μM (*p* = 0.005) conditions. After 2 days, only the 150 μM led to a decrease of 22% in the DNA content compared to CTR (*p* = 0.004). By day 3, no significant differences were observed between the tested conditions (Figure 2C).

**Figure 3:**
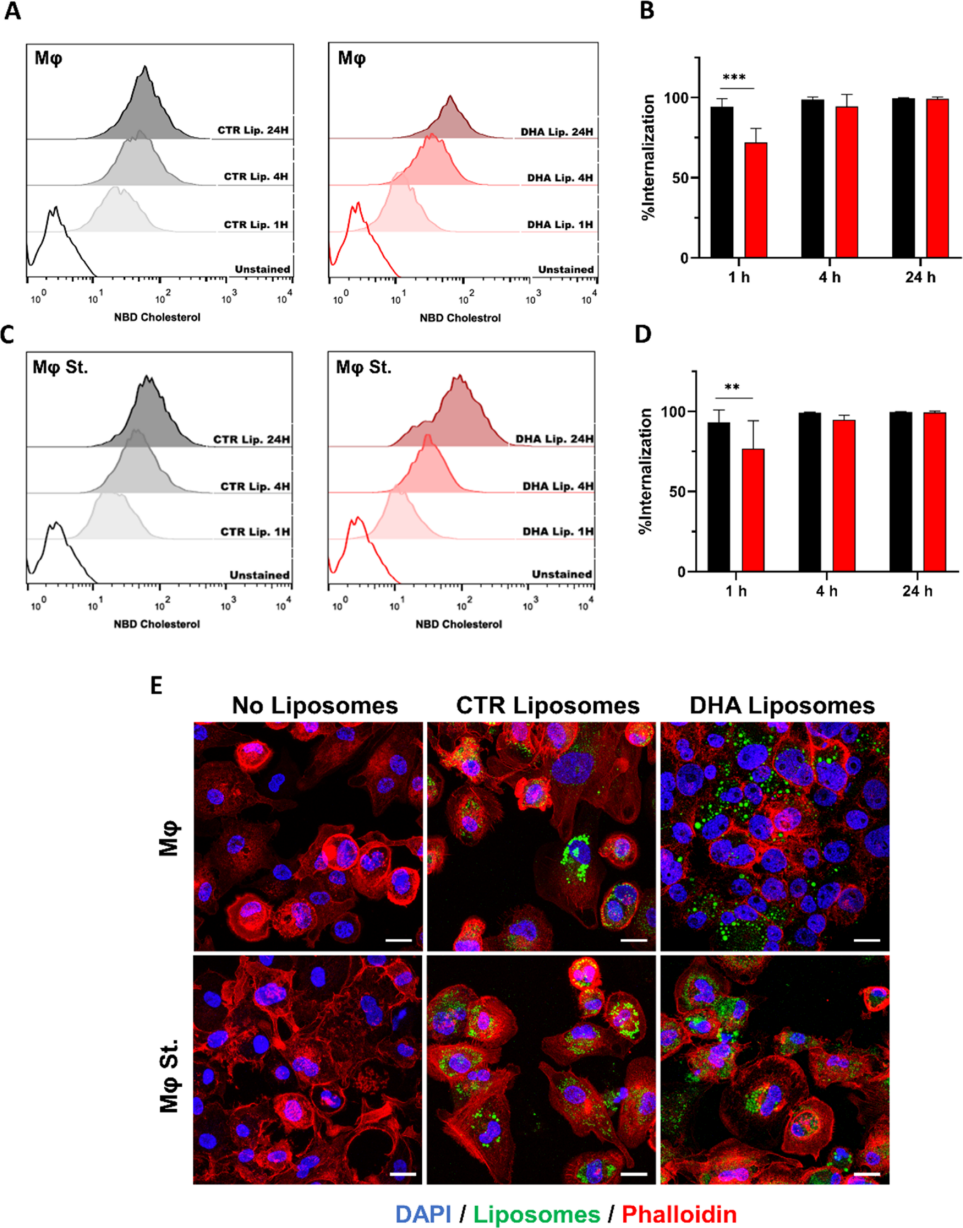
Liposomes uptake by macrophages Flow cytometry histograms of macrophages (Mφ (A)) and stimulated macrophages (Mφ St (C)) incubated for 1, 4, and 24 h with 500 μM of control (CTR) and docosahexaenoic acid (DHA) fluorescent labeled liposomes. Quantification of internalization percentage over time (black, CTR liposomes; red, DHA liposomes; (B, D)). Confocal microscopy images of Mφ and Mφ St, after 4 h of incubation with CTR and DHA liposomes blue: DAPI; red: phalloidin; green: liposomes; (E). Statistically significant differences between conditions are represented as **p < 0.01 and ***p < 0.001.

The targeting and internalization of liposomes by macrophages and stimulated macrophages were also assessed over time. Flow cytometry analyses revealed that both CTR and DHA liposomes were uptaken by macrophages in a time-dependent manner. Indeed, an increase in the fluorescence signal (NBD cholesterol) was observed over time. Within the first hour, CTR liposomes were significantly more internalized than DHA liposomes by macrophages (*p* < 0.001; Figure 3A,B) and stimulated macrophages (*p* < 0.004; Figure 3C,D). No significant differences were observed between the liposome nanoformulations after 4 and 24 h of incubation with more than 90% of the analyzed cells presenting a positive fluorescent signal (Figure 3B,D). Furthermore, confocal microscopy pictures (Figure 3E) showed the presence of CTR and DHA liposomes in the cytoplasm of macrophages and stimulated macrophages after 4 h of incubation.

**Figure 4:**
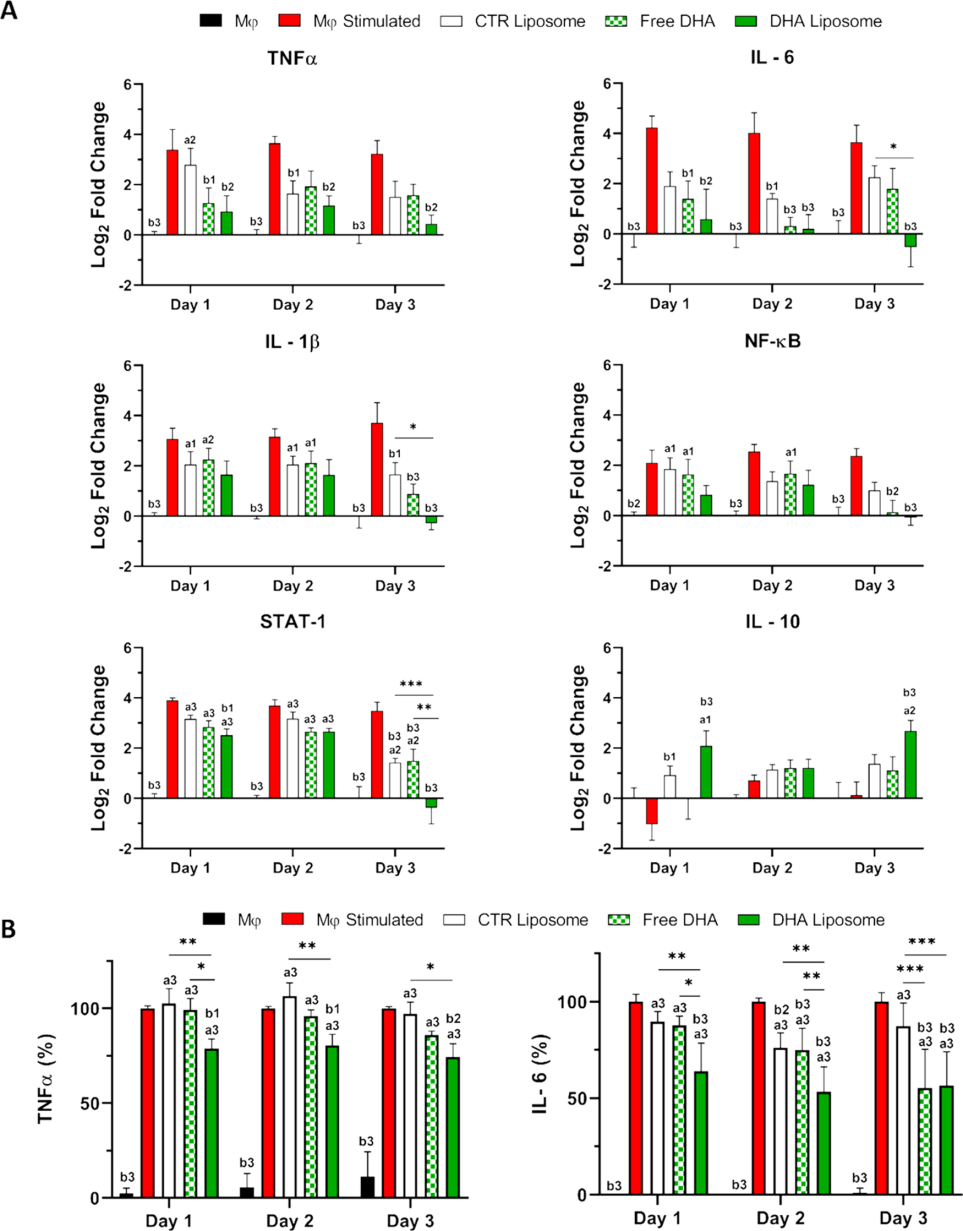
Inflammatory mediation of macrophages by DHA liposomes. Expression of immunomodulatory genes by macrophages was analyzed by quantitative polymerase chain reaction (qPCR). Nonstimulated macrophages (Mφ) and stimulated macrophages (Mφ stimulated) were used as control conditions to access the inflammatory gene expression and cytokines secretion. Mφ stimulated were treated for 1, 2, or 3 days with free DHA, CTR liposomes, and DHA liposomes. Gene expression is presented as the logarithmic fold change relative to Mφ condition expression and is normalized against glyceraldehyde-3-phosphate dehydrogenase (GAPDH) (A). Secretion of tumor necrosis factor α (TNFα) and Interleukin 6 (IL-6) by macrophages was analyzed by enzyme-linked immunosorbent assay (ELISA) assay, relative to Mφ stimulated condition, after incubation with free DHA, CTR liposomes, and DHA liposomes (B). Data were analyzed by 2-way ANOVA and Tukey’s multiple comparations test: a1 (*p* < 0.05), a2 (*p* < 0.01), and a3 (*p* < 0.001) denote significant differences compared to Mφ, and b1 (*p* < 0.05), b2 (*p* < 0.01) and b3 (*p* < 0.001) are differences relative to stimulated Mφ. The remaining differences between conditions are represented as *p < 0.05, **p < 0.01, and ***p < 0.001.

The effect of DHA liposomes on the expression of inflammation markers was assessed in the macrophages. Based on the cytocompatibility and cell uptake results, stimulated macrophages were incubated with 50 μM DHA incorporated or not in liposomes. The expression of inflammatory-associated genes was assessed by qPCR for 3 days and macrophages without stimulation and CTR liposomes were used as control conditions (Figure 4A). The stimulation of macrophages with LPS and IFN-γ led to a significant increase in the gene expression of inflammatory-associated molecules (*TNFα*, *IL-6*, *IL-1β*, nuclear factor kappa-light-chain-enhancer of activated B cells- *NF-κB*, and signal transducer and activator of transcription 1-*STAT-1*) between 2- and 5-fold relative to nonstimulated macrophages at all time points. A significant decrease in the expression of these pro-inflammatory genes relative to stimulated macrophages was obtained after treatment with DHA liposomes. In most of the analyzed genes, their expression was reduced to similar levels in macrophages without stimulation (Figure 4A). Free DHA and CTR liposomes were also able, although not so noticeable, to reduce the expression of the genes of the inflammatory mediators *TNFα*, *IL-6*, and *IL-1β*. The difference between CTR liposomes and DHA liposomes was more noticeable at day 3, with higher fold differences between the overall expression of the genes observed at that time point. Regarding the expression of *IL-10*, an overall opposite trend was found. Indeed, there was an increased expression of this gene on day 1 and day 3 after treatment with DHA liposomes. Conversely, no differences were observed between free DHA and stimulated macrophages, and only at day 1 significant variance was found between CTR liposome and control conditions (Figure 4A).

Regarding the secretion of pro-inflammatory cytokines secreted by macrophages, a significant reduction was observed after treatment with DHA liposomes during the 3 days. A reduction of 21.10, 19.42, and 25.72% in the amount of TNFα was observed after treatment of stimulated macrophages with DHA liposomes on days 1, 2, and 3, respectively (Figure 4B). Moreover, differences between CTR liposomes and DHA liposome treatment were observed on day 1 (*p* = 0.009), day 2 (*p* = 0.003), and day 3 (*p* = 0.012). Conversely, differences between free DHA and DHA liposome conditions were found only on day 1 (*p* = 0.033). Regarding the secretion of IL-6 by stimulated macrophages, DHA liposomes successfully reduced their amount by over 35% at all time points (*p* < 0.001). The exposure to free DHA was also able to reduce the secretion of IL-6 on day 2 and day 3 (*p* < 0.001). Furthermore, significant differences were found comparing the CTR and DHA liposome conditions on the 3 analyzed days (Figure 4B).

**Figure 5:**
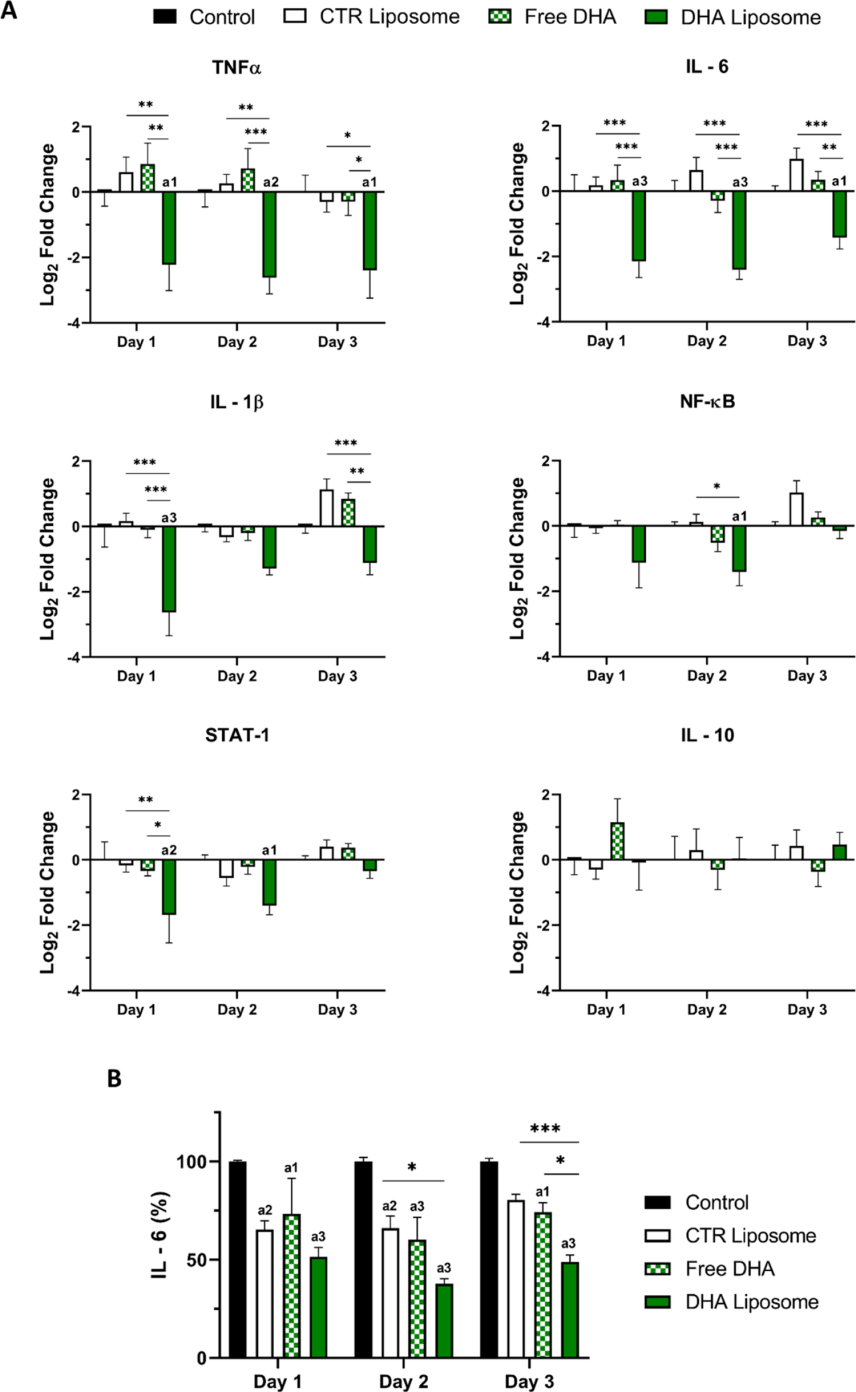
Inflammatory mediation of GBM by DHA liposomes. Expression of immunomodulatory genes by U87 cells after treatment for 1, 2, or 3 days with free DHA, CTR liposomes, and DHA liposomes analyzed by quantitative polymerase chain reaction (qPCR). Gene expression is presented as logarithmic fold change relative to untreated condition basal expression (control) and is normalized against glyceraldehyde-3-phosphate dehydrogenase GAPDH (A). Secretion of interleukin 6 (IL-6) by U87 cells was analyzed by enzyme-linked immunosorbent assay (ELISA) assay, relative to control condition, after incubation with free DHA, CTR liposomes, and DHA liposomes (B). Data were analyzed by 2-way ANOVA and a Tukey’s multiple comparations test: a1 (*p* < 0.05), a2 (*p* < 0.01), and a3 (*p* < 0.001) denote significant differences compared to control. Other statistically significant differences between conditions are represented as *p < 0.05; **p < 0.01; ***p < 0.001.

The expression of inflammatory-associated genes was also assessed in GBM cells treated with CTR liposomes, free DHA, and DHA liposomes. A significant decrease in the relative gene expression of *TNFα*, *IL-6*, and *IL-1β* after treatment with DHA liposomes during the 3 days was observed, compared to the control (Figure 5A). Moreover, no significant differences were observed when cells were treated with CTR liposomes and free DHA. Regarding the expression of *NF-κB* and *STAT-1*, DHA liposomes also significantly reduced the expression of these genes relative to the control conditions. Control liposomes and free DHA conditions did not reveal significant effects in the expression of the analyzed genes. Additionally, no differences were observed regarding the expression of *IL-10* under either of the conditions. Considering the importance of IL-6 in the modulation of TME, the secretion of this cytokine by GBM cells was analyzed by an ELISA assay (Figure 5B). A significant decrease in the amount of IL-6 was observed during the 3 days of incubation with CTR liposome, free DHA, and DHA liposome. The DHA liposome revealed the ability to induce an accentuated decrease in the secretion of IL-6 (reduction of 48.5, 62.28, and 51.06% on days 1, 2, and 3, respectively, compared to the positive control condition). At day 3, significant differences were also observed between the DHA liposome and CTR liposome (*p* = 0.009) or free DHA (*p* = 0.05).

## Discussion

In this work, DHA liposomes were successfully produced through a micromixer chip that allows for the precise mixing of miscible organic and aqueous phases, leading to the synthesis of liposomes by controlled precipitation within its channels.^38^ Based on previous research conducted by our group, the flow rate ratios and flow rates were set to produce this type of nanoparticle with a size below 100 nm (Figure 1A–C). Importantly, the incorporation of DHA in the liposomal formulation did not significantly change the physiochemical properties of the liposomes, namely, morphology, size distribution, stability, and surface charge (Figure 1). Although specific nanoparticle features for accumulation in the tumor site are still being investigated, key properties, like the hydrodynamic size of approximately 100 nm, rod-shaped architecture, and neutral/slightly negative charge favor their accumulation at the tumor.^39,40^ Thus, efforts were performed to produce liposomes with these features. Indeed, the developed homogeneous (PDI < 0.2) and stable (Figure 1D) suspensions of DHA liposomes presented diameters of ≈86 nm (Figure 1C) and a negative surface charge (Figure 1E). Moreover, the overall size of these particles allows for reducing some of the hepatic uptake and clearance rate.^41^ Additionally, it allows for the exploitation of the enhanced permeability and retention (EPR) effect in the highly compromised and vascular irrigated GBM mass and diffusion across the TME.^42^ The AFM analyses revealed a spherical-like morphology (Figure 1B), which also favors their biodistribution to tumor sites. Moreover, the interfacial properties conferred by the slightly negative ζ-potential of the liposomes can improve the nanocarriers’ half-lives in circulation, which can translate to improved accumulation of the liposomes within the tumor.^39^

In nanomedicine strategies for cancer treatment, the ability of nanocarriers to evade phagocytic cell clearance is a critical characteristic.^43^ However, in this study and considering the role of a large number of key phagocytic cells in the TME, the uptake of DHA liposomes by macrophages can have positive implications. To establish the concentration to be used in the internalization experiments, the cytocompatibility of the generated liposomes was first assessed. Considering the metabolic activity and DNA concentration data (Figure 2), a concentration of 500 μM liposomes or liposomes containing 50 μM of DHA were used for the following experiments. DHA concentrations in this order of magnitude are sufficient to trigger apoptosis in GBM cells.^22^ Interestingly, at similar or lower concentrations of DHA liposomes, we observed an increased metabolic activity that could be associated with phagocytic events following their exposure. During phagocytosis, an increase in glycolysis occurs to support the energy necessities of macrophages,^44^ potentially leading to the observed rise in the metabolic activity presented in this study. Indeed, liposomes were quickly uptaken by control and stimulated macrophages (Figure 3), common among lipidic nanocarriers,^33^ which can result in their degradation and subsequent release of DHA in the intracellular environment. The metabolization of DHA can result in bioactive derivatives that will reduce the inflammatory milieu of GBM.^27^ Thus, this study focused on modulating the inflammatory mediation performed by macrophages rather than eliminating them. Moreover, together with the cancer cells’ apoptosis triggered by DHA, as already demonstrated,^22^ these liposomes can have a synergistic effect in GBM therapy. By targeting cancer cells and reducing the functional role of macrophages in GBM, the progression of the tumor development can be significantly reduced. Indeed, the paracrine and autocrine circuits between myeloid and GBM cells have been studied due to their influence on the progression of the tumor.

Aberrant inflammation is a significant trait in GBM, which not only endows tumor cells with an immune evasion ability but also exacerbates tumor proliferation, invasion, and relapse.^45,46^ In this study, DHA liposomes were able to successfully mitigate the inflammatory profile in stimulated macrophages (Figure 4) and GBM cells (Figure 5), by reducing *IL-6*, *IL-1β*, and *TNFα* gene expression. Moreover, the secretion of IL-6 and TNFα was also significantly reduced, showing the anti-inflammatory efficacy of DHA liposomes (Figures 4 and 5). Previous studies have explored the impact of DHA on inflammation, although most of them are primarily focusing on its dietary intake.^47^ Its anti-inflammatory effect is mainly attributed to the inhibition of eicosanoid synthesis from arachidonic acid (AA), which is typically highly present in cells prone to undergo neoplastic transformation.^48^ For instance, in breast cancer mice models, dietary DHA consumption led to a reduction of AA and its major subproduct prostaglandin E_2_, resulting in the inhibition of tumor cell growth and metastization.^49^ In other reports, a DHA dose-dependent effect in the downregulation of cell cycle and inflammatory-associated genes was observed in LPS-stimulated macrophages.^50^ In the work discussed here, DHA liposomes were significantly more efficient to downregulate the expression of main pro-inflammatory cytokine-associated genes in macrophages (Figure 4) and GBM cells (Figure 5), compared with the same concentration of free DHA. Consequently, the anti-inflammatory properties of DHA were kept, and by incorporating this fatty acid in liposomes, the biological activity was significantly improved. These results can be explained by the different cellular uptake pathways between free DHA and DHA liposomes, and by the protection of DHA against degradation, since it is highly susceptible to fast oxidation,^51^ that the liposomal formulation confers. Interestingly, the CTR liposomes (without DHA) decreased, although to a significantly lower extent, the expression of some of the inflammatory-associated genes. These results can be associated with the composition of the liposomes, more precisely with the presence of PC, which has been linked to anti-inflammatory properties.^52^

Some strategies have been explored to mitigate the effect in the tumors of the overexpressed IL-1β, IL-6, TNFα, and other cytokine precursor genes that are directly correlated with a poor prognosis in patients.^53,54^ Particularly, among the several inflammatory mediators that regulate GBM, the upregulation of IL-6 has been strongly correlated with glioma grade and overall decreased patient survival.^55^ The upregulation of the gene and production of IL-6 occurs in several cells of the TME, including macrophages and GBM cells, in response to an inflammatory stimulus (*e.g.*, trauma, cancer). IL-6 is associated with several GBM hallmarks, including proliferation, invasion, angiogenesis, and resistance to cell death.^56^ Most of these phenomena occur through the triggering of the Janus kinase/signal transducer and activator of transcription 3 (JAK-STAT-3) pathway that is highly active in GBM, being thus a target of several developed drugs.^56^ For instance, tocilizumab, a humanized antibody that blocks IL-6 receptors, was able to inhibit cell proliferation in GBM cell lines.^57^ The upregulation of IL-6 is also highly associated with the expression and production of TNFα by macrophages and cancer cells. This cytokine is associated with GBM cell invasion and proliferation by regulating and activating p65 (subunit of the NF-κB transcription factor complex) and protein kinase B (PKB, also known as Akt) signaling pathways.^58,59^ In this way, by downregulating these main pro-inflammatory cytokines and affecting the inflammatory milieu, DHA liposomes can directly impair GBM growth and progression.

In this study, a significant decrease in the expression of NF-κB in macrophages and cancer cells after treatment with DHA formulations was also observed. Similar findings were obtained in other studies with ω3 fatty acids.^60^ Interestingly, in the last years, NF-κB has emerged as a driver of multiple aspects of gliomagenesis and resistance to treatment.^61,62^ The NF-κB can be activated by IL-1β, leading to persistent stimulation of pro-inflammatory genes.^63^ Among others, the activation of NF-κB and STAT-3 pathways in GMB cells regulates the pro-tumoral effect of the cytokines TNFα and IL-6.^15,64^ The targeting of this family of transcription factors, with nonsteroidal anti-inflammatory drugs or antibodies, successfully suppressed the growth and chemoresistance of GBM cells in preclinical studies.^65^ Furthermore, the use of DHA liposomes was found to result in the downregulation of STAT-1. This downregulation has the potential to diminish the aggressiveness of GBM cells by inhibiting epithelial-mesenchymal transition, which is mediated by the wnt/β-catenin signaling pathway.^66,67^ The work developed here shows that encapsulating DHA in liposomes enhances its stability and effectiveness as a therapeutic molecule. Consequently, DHA can inhibit the synthesis of key pro-inflammatory mediators, such as IL-6 and TNFα, thereby modulating the inflammatory state in both macrophages and GBM cells.

## Conclusions

In this study, liposomes incorporating DHA were successfully developed by using a microfluidic synthesis methodology. These DHA liposomes were quickly internalized by stimulated and nonstimulated macrophages, without compromising the viability and proliferation of these myeloid cells. Remarkably, it was observed that DHA liposomes were more efficient in reducing the expression of key inflammatory genes and cytokines in stimulated macrophages and GBM cells compared to the free DHA, highlighting the potential of this ω3 fatty acid nanoformulation for the treatment of GBM. The nanomedicine platform developed in this work validates the utilization of DHA as an anticarcinogenic and anti-inflammatory agent, offering enhanced efficiency through its incorporation in stable and reproducible liposomes.

## Supporting Information Available

RNA concentration and purity of all samples (Table S1) (PDF)
