# Supplementary material for: Microfluidic-Derived Docosahexaenoic Acid Liposomes for Targeting Glioblastoma and Its Inflammatory Microenvironment
Source: ACS Appl Mater Interfaces. 2024 Jul 23;16(31):40543–54. doi: 10.1021/acsami.4c01368 (PMC11310905; doi:10.1021/acsami.4c01368)
Supplement: Supplementary file 1 — am4c01368_si_001.pdf [file am4c01368_si_001.pdf]

Supporting information

Microfluidic-derived docosahexaenoic acid liposomes for targeting glioblastoma and its inflammatory microenvironment

Daniel Mendanha [1,2], Marta R. Casanova [1,2], Sara Gimondi [1,2], Helena Ferreira [1,2], Nuno M.Neves [1,2,]*

[1]3B's Research Group, I3Bs – Research Institute on Biomaterials, Biodegradables and Biomimetics, University of Minho, Headquarters of the European Institute of Excellence on Tissue Engineering and Regenerative Medicine, AvePark, Parque de Ciência e Tecnologia, Zona Industrial da Gandra, 4805-017 Barco, Guimarães, Portugal

[2]ICVS/3B's-PT Government Associate Laboratory, 4805-017 Barco, Braga/Guimarães, Portugal

*Corresponding author: nuno@i3bs.uminho.pt

Table S1. RNA concentration (ng/μL) and purity determined for each sample.

**Sample ID**			**RNA Concentration (ng/μL)**	**Purity (A260/A280)**
N1	Day 1	Mφ_1	65.254	1.85
		Mφ_2	16.889	1.75
		Mφ_3	14.775	1.87
		Mφ Stimulated_1	11.592	1.70
		Mφ Stimulated_2	21.996	1.87
		Mφ Stimulated_3	21.45	1.89
		CTR Liposome_1	15.621	1.78
		CTR Liposome_2	26.795	1.73
		CTR Liposome_3	28.083	1.83
		Free DHA_1	80.378	1.86
		Free DHA_2	41.756	1.81
		Free DHA_3	96.055	1.85
		DHA Liposome_1	35.003	1.77
		DHA Liposome_2	28.849	1.83
		DHA Liposome_3	24.435	1.81
	Day 2	Mφ_1	83.015	1.84
		Mφ_2	44.783	1.89
		Mφ_3	77.39	1.84
		Mφ Stimulated_1	38.909	1.82

		Mφ Stimulated_2	28.26	1.73
		Mφ Stimulated_3	63.305	1.82
		CTR Liposome_1	21.289	1.88
		CTR Liposome_2	61.222	1.82
		CTR Liposome_3	57.816	1.82
		Free DHA_1	64.171	1.72
		Free DHA_2	32.707	1.74
		Free DHA_3	61.555	1.81
		DHA Liposome_1	59.004	1.83
		DHA Liposome_2	38.684	1.80
		DHA Liposome_3	76.425	1.80
	Day 3	Mφ_1	55.284	1.88
		Mφ_2	43.298	1.86
		Mφ_3	58.051	1.88
		Mφ Stimulated_1	67.739	1.81
		Mφ Stimulated_2	40.293	1.85
		Mφ Stimulated_3	68.298	1.89
		CTR Liposome_1	45.723	1.92
		CTR Liposome_2	37.103	1.82
		CTR Liposome_3	55.454	1.89
		Free DHA_1	37.857	1.82
		Free DHA_2	34.379	1.78
		Free DHA_3	41.692	1.86
		DHA Liposome_1	36.634	1.8
		DHA Liposome_2	29.511	1.72

		DHA Liposome_3	26.395	1.81
N2	Day 1	Mφ_1	5.24	1.88
		Mφ_2	5.43	1.86
		Mφ_3	6.69	1.80
		Mφ Stimulated_1	9.68	1.83
		Mφ Stimulated_2	7.79	1.80
		Mφ Stimulated_3	9.38	1.82
		CTR Liposome_1	20.97	1.84
		CTR Liposome_2	8.32	1.86
		CTR Liposome_3	9.59	1.83
		Free DHA_1	8.84	1.74
		Free DHA_2	8.35	1.81
		Free DHA_3	9.51	1.88
		DHA Liposome_1	6.18	1.82
		DHA Liposome_2	6.46	1.87
		DHA Liposome_3	6.5	1.87
	Day 2	Mφ_1	6.21	2.01
		Mφ_2	7.48	1.88
		Mφ_3	7.81	1.88
		Mφ Stimulated_1	5.8	1.82
		Mφ Stimulated_2	7.42	1.86
		Mφ Stimulated_3	5.05	1.89
		CTR Liposome_1	6.06	1.80
		CTR Liposome_2	5.59	1.86

		CTR Liposome_3	4.91	1.85
		Free DHA_1	5.12	1.81
		Free DHA_2	7.14	1.81
		Free DHA_3	7.68	1.80
		DHA Liposome_1	5.91	1.82
		DHA Liposome_2	6.16	1.83
		DHA Liposome_3	5.85	1.89
	Day 3	Mφ_1	7.49	1.88
		Mφ_2	16.04	1.89
		Mφ_3	26.28	1.89
		Mφ Stimulated_1	4.03	1.86
		Mφ Stimulated_2	5.01	1.87
		Mφ Stimulated_3	5.3	1.86
		CTR Liposome_1	6.11	1.86
		CTR Liposome_2	4.19	1.88
		CTR Liposome_3	4.07	1.88
		Free DHA_1	4.9	1.85
		Free DHA_2	20.21	1.80
		Free DHA_3	4.1	1.89
		DHA Liposome_1	6.59	1.83
		DHA Liposome_2	7.45	1.83
		DHA Liposome_3	86.64	1.87
N3	Day 1	Mφ_1	45.62	1.89
		Mφ_2	32.07	1.86
		Mφ_3	12.6	1.81

		Mφ Stimulated_1	30.74	1.82
		Mφ Stimulated_2	15.63	1.86
		Mφ Stimulated_3	12.36	1.89
		CTR Liposome_1	6.71	1.89
		CTR Liposome_2	16.81	1.86
		CTR Liposome_3	14.24	1.87
		Free DHA_1	9.79	1.84
		Free DHA_2	13.95	1.83
		Free DHA_3	9.62	1.80
		DHA Liposome_1	14.62	1.80
		DHA Liposome_2	11.76	1.85
		DHA Liposome_3	8.91	1.81
	Day 2	Mφ_1	9.76	1.89
		Mφ_2	12.1	1.89
		Mφ_3	24.74	1.81
		Mφ Stimulated_1	14.33	1.86
		Mφ Stimulated_2	12.67	1.80
		Mφ Stimulated_3	10	1.80
		CTR Liposome_1	17.29	1.83
		CTR Liposome_2	10.13	1.86
		CTR Liposome_3	9.3	1.89
		Free DHA_1	7.75	1.85
		Free DHA_2	10.26	1.85
		Free DHA_3	4.03	1.80
		DHA Liposome_1	9.5	1.83

		DHA Liposome_2	9.02	1.80
		DHA Liposome_3	8.65	1.86
	Day 3	Mφ_1	5.83	1.87
		Mφ_2	5.72	1.89
		Mφ_3	24.56	1.83
		Mφ Stimulated_1	4.31	1.84
		Mφ Stimulated_2	7.11	1.88
		Mφ Stimulated_3	6.24	1.86
		CTR Liposome_1	5.98	1.83
		CTR Liposome_2	6.65	1.80
		CTR Liposome_3	12.11	1.85
		Free DHA_1	9.05	1.88
		Free DHA_2	4.51	1.81
		Free DHA_3	6.71	1.81
		DHA Liposome_1	12.02	1.80
		DHA Liposome_2	4.11	1.81
		DHA Liposome_3	4.16	1.80
